# Supplementary material for: Complexome profiling of the Chlamydomonas psb28 mutant reveals TEF5 as an early PSII assembly factor
Source: Plant Cell. 2025 Mar 17;37(6):koaf055. doi: 10.1093/plcell/koaf055 (PMC12164590; doi:10.1093/plcell/koaf055)
Supplement: koaf055_Supplementary_Data [file koaf055_supplementary_data.zip › psb28_interactive.zip!/plots/intensity/qualityPlots/peptides/TGD2_Cre16.g694400.t1.2.html]

### Cre16.g694400.t1.2

Trivial name: TGD2  
SeqCoverage [median]: 35.40  
Mass: 34.61 kDa  
  
 

| Sequence | MissedCleavage | Mass [Da] | wtIntensity | muIntensity | Score |
| --- | --- | --- | --- | --- | --- |
| AAPLVSSAAELVEQLTPLMAELR | 0 | 2408.30 | 828.98 | 0.00 | 72.99 |
| GGGLVGNIEALTR | 0 | 1255.69 | 2719.68 | 0.00 | 89.83 |
| GVQVGQVLAVKPSLER | 1 | 1678.97 | 9126.03 | 19442.44 | 124.09 |
| LQGSVLTEDNVR | 0 | 1329.69 | 3132.51 | 8086.08 | 195.85 |
| QGLFGSIAK | 0 | 919.51 | 846.23 | 0.00 | 79.47 |
| RLQGSVLTEDNVR | 1 | 1485.79 | 0.00 | 1852.51 | 63.66 |
| SLSDYGIGKK | 1 | 1066.57 | 4032.67 | 5304.87 | 79.15 |
| TAADAAADIR | 0 | 973.48 | 4423.63 | 5723.20 | 130.66 |
| TAADAAADIRR | 1 | 1129.58 | 5101.64 | 12786.66 | 90.05 |
| TLVQALSR | 0 | 886.52 | 7583.90 | 15657.25 | 131.06 |
|  |  |  | 37795.3 | 68853.0 |  |
